# Chromosome Abnormalities and Viability of Vitrified Eight-Cell Mouse Embryos at Presence of Two Different Cryoprotectants at Different Storage Durations

**Published:** 2013-02-20

**Authors:** Shabnam Zarei Moradi, Anahita Mohseni Meybodi, Hamid Gourabi, Hossein Mozdarani, Zahra Mansouri

**Affiliations:** 1. Department of Genetics at Reproductive Biomedicine Research Center, Royan Institute for Reproductive Biomedicine, ACECR, Tehran, Iran; 2. Department of Medical Genetics, School of Medical Sciences, Tarbiat Modares University, Tehran, Iran

**Keywords:** Chromosome Abnormality, Cryopreservation, Mouse Embryo, Viability

## Abstract

**Objective:**

Experiments were conducted to find the differences between post-thaw viability and chromosome aberrations in eight-cell mouse embryos at presence of dimethyl sulfoxide (DMSO) and 1, 2-propanediol (PROH) as croprotectants in different storage durations.

**Materials and Methods:**

In this case-control study, a total number of 720 mouse embryos from about 250 NMRI mice were vitrified with 30% PROH or DMSO; each diluted with a solution containing 30% ficol plus 0.5 M sucrose. Embryos were exposed to the solutions for 0.5 minute at 25℃ followed by cooling in liquid nitrogen, then after appropriate storage duration, they were rapidly warmed. Besides, there were 100 mouse embryos for each cryoprotectant group (totally 200 embryos) as control. Embryo survival was assessed by *in vitro* development, and chromosome abnormalities were analyzed by Giemsa staining.

**Results:**

The proportion of mitotic abnormalities in PROH/DMSO vitrified embryos was significantly higher than unfrozen control group. This was confirmed also by a reduced viability of the embryos as judged by a culture at the blastocyst stage (p<0.05 in all test groups).

**Conclusion:**

It can be deduced that long term cryopreservation may result in chromosomal abnormalities and/or low viability.

## Introduction

Embryo cryopreservation followed by thawing and transferring into the uterus, offers several advantages in assisted reproductive technology (ART) programs. Storing embryos in liquid nitrogen has become as a facility to transfer a limited number of embryos on consecutive occasions. This method can provide an increased pregnancy rate and reduce the risk of multiple gestations and ovarian hyperstimulation syndrome (1, 2). As multiple gestation, with its innate risk for undesirable outcome, remains a great concern in ART treatments, the single-embryo transfer (SET) strategy has become an accepted method step by step (3, 4). One consequence of SET is an increase in availability of supernumerary embryos for freezing (5), resulting in more children born after cryopreservation, and a decrease in multiple pregnancies (6). Cryopreservation will also increase the chance of pregnancy in a natural cycle without additional ovarian stimulation and oocyte retrieval (7). It is noted that vitrification is a novel cryopreservation method for mammalian blastocysts and becomes a routine procedure in infertility clinics, but any strong conclusions about the safety of these techniques have not been provided yet (8-11).

There are some reports showed freezing and thawing significantly either reduce embryo viability (12, 13) or delay embryo development (14, 15); however, the question of whether or not freeze-thawing method causing genetic damage has not been satisfactorily answered. It was suggested that depolymerization of microtubules by cryoprotectants or by cooling may prevent the normal separation of sister chromatids through which non-disjunction may lead to aneuploidy (1). If any aberrant changes are induced in the DNA of these eight-cell embryos by extraneous factors, their descendants may carry chromosome anomalies.

Although cryopreservation of embryos is part of most *in vitro* fertilization (IVF) programs, only limited studies on perinatal revealing the outcome of children born after replacement of the cryopreserved embryos are available, at present time (8, 16-23).

In some studies major chromosomal abnormalities such as trisomy of chromosome 13, 18 and 21, have been observed in children born from frozen embryos (17, 19, 24). On the other hand, a comparison of children conceived from frozen-thawed embryos with those born normally or from fresh IVF cycles showed a similar or even decreased incidence of congenital abnormalities after cryopreservation (16, 25, 7).

Our previous research on mouse embryos has shown that vitrification may cause some chromosomal damage (26). Also, another report has revealed increased mitotic crossing over in mouse embryos after cryopreservation (27). Based on these findings and observation of chromosome abnormalities in our previous study, we aimed to explore the link between chromosomal status and viability at presence of two different cryoprotectants in different storage durations.

## Materials and Methods

### Collection of eight – cell mouse embryos

About 250 female NMRI mice (Pasteur Institute, Tehran, Iran) aged 6-8 weeks were super ovulated with an intraperitoneal injection of 10 IU of pregnant mare serum gonadotropin (PMSG) (Intervet, Netherlands), followed by another intraperitoneal injection of 10 IU of human chorionic gonadotropin (hCG,Organon, Netherlands) in 48 hours later. The females were mated singly with 2 adult males from the same strain. After 66-68 hours of mating, the mice were killed by cervical dislocation and eightcell embryos were flushed from their oviducts into T6 medium. It should be mentioned that about 1000 embryos were subjected to this procedure.

### Vitrification solutions

PB1 medium: Dulbecco’s phosphate-buffered saline (PBS) includes: CaCl_2_, 2H_2_O (0.132 µg/ml), KCl (200 µg/ml), KH_2_PO_4_ (200 µg/ml), MgCl_2_ (100 µg/ ml), NaCl (8 mg/ml), Na_2_HPO_4_ (1.15 mg/ml)], glucose (5.56 mmol/L), pyruvate (0.33 mmol/L), penicillin G (100 IU/ml), streptomycin (100 µg/ml), and bovine serum albumin (BSA) (3 mg/ml). *Sucrose solution*: PB1 medium contains 0.5 mol/L sucrose.

*ficol-sucrose (FS) solution* is prepared as follows: Ficol 70 (Mol.Wt. 70, 000) is added to 35.1 ml filter sterilized PB1medium. Leave it at room temperature until ficol disappears. Then, sucrose (8.56g) is added followed by adding 105 mg of BSA. All of the ingredients must be combined and thoroughly dissolved.

*PROH FS 30%* and *DMSO FS 30%* are prepared as follows: 30% PROH or DMSO is added to FS solution to make 30% (v/v) DMSO or PROH vitrification solution with the final concentration of 21% (w/v) ficol and 0.35M sucrose (28).

### Freezing-thawing

Healthy intact eight-cell embryos in each weekly flushing were equally divided into 6 different groups based on storage durations, including: 24-hour, 1-week, 2-week, 1-month, 3-month and 6-month groups. It should be mentioned that there was 2 control groups, each contains 100 embryos: the first group was assessed for viability rate up to blastocyst stage, and the second one has been analyzed from chromosomal point of view (polyploidy or aneuploidy). PROH and DMSO were applied as the cryoprotectants for all above-mentioned test groups. Every 10 embryos were directly suspended in a vitrification solution and loaded into a 0.25ml straw, at room temperature (25℃). The configuration of the straw was described, previously (29). After exposure of the embryos to the vitrification solution for 30 seconds, the straws were plunged into liquid nitrogen. After appropriate storage durations (24 hours, 1 and 2 weeks, as well as 1, 3 and 6 months), embryos were thawed. Straws were taken out of liquid nitrogen and immediately plunged into water at 25℃. After five seconds, the straws were removed from the water, quickly wiped dry and the contents of the straw were expelled into a watch glass containing sucrose solution, by cutting two ends of the straw by scissors. The embryos were then pipetted into fresh T6 medium prepared under paraffin oil in a culture dish.

### Assessment of post-thaw viability of embryos

Embryos recovered after vitrification were washed and cultured in T6 medium under paraffin oil in a culture dish in 5% CO_2_ incubator at 37℃. Then, the survival of embryos was assessed by their ability to develop to the blastocysts in culture dish.

### Assessment of chromosome abnormalities

After four-six hours in T6 medium, the embryos were exposed to T6 medium containing colcemid for 18 hours, and then embryos were plunged in Tyrode’s solution acid for four seconds in three steps to slenderize zona pellucida. In the next step, embryos were placed in hypotonic solution (sodium citrate 1%) until swollen (three-five minutes). The swollen embryos were individually placed on a clean chilled glass microscope slide with a minimal amount of solution, and then spread using fixation solution described previously by Tarkovski (30). The slides were stained in Giemsa (3%), and examined under oil immersion microscope (×100) for numerical chromosome analysis.

### Statistical analysis

Survival rates of test and control groups (PROH test group, DMSO test group and control group) were analyzed using the Chi square test (Χ^2^). Fisher’s exact test and chi square were used to compare chromosome abnormalities between groups. Statistically significance was defined as p<.05. Statistical analysis was performed with Statistical Package for the Social Sciences (SPSS, Chicago).

## Results

### Viability

**Fig 1:**
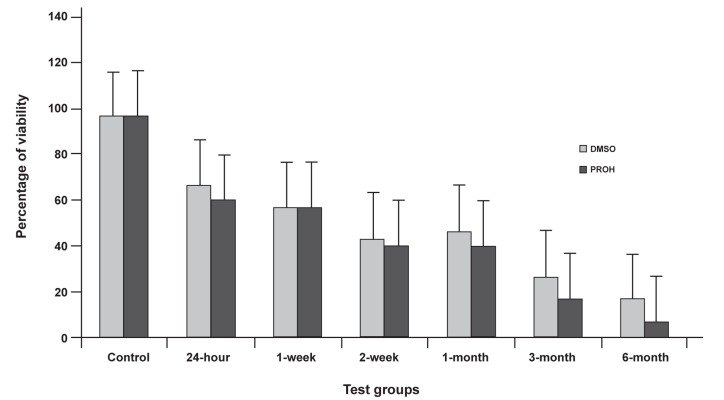
Percentage of viability at the presence of DMSO and PROH as cryoprotectant for various storage durations . Error bars show SE of mean values calculated for data obtained from different samples.

Cryopreservation impaired the *in vitro* development of the embryos, as demonstrated by lower rate of the blastocyst formation observed both for PROH and DMSO vitrified embryos compared with the control group (Fig 1). The viability of PROH vitrified embryos after cryopreservation was lower than DMSO (Fig 1, Table 1).

**Table 1:** The Results of eight-cell mouse embryos viability vitrified in DMSO/PROH solution after various storage durations


Test groups	No. of Embryos			P value		Vitrified	Survived (%)	Degenerated	

Control	100	97(97)	3	<0.05
24-hour	30	20(66.7)	10	<0.05
	30	18(60)	12	
1-week	30	17(56.6)	13	<0.05
	30	17(56.6)	13	
2-week	30	13(43.3)	17	<0.05
	30	12(40)	18	
1-month	30	14(46.7)	16	<0.05
	30	12(40)	18	
3-month	30	8(26.6)	22	<0.05
	30	5(16.7)	25	
6-month	30	5(16.7)	25	<0.05
	30	2(6.6)	28	


As shown in table 1 as well as figure 1, by increasing storage duration, viability rate decreased. For example, in 24-hour group vitrified by DMSO/ PROH, viability rates were 66.7% and 60%, and for 6-month group were 16.7% and 6.6%, respectively.

On the other hand, no significant difference can be seen in the viability rates of 2-week groups of both cryoprotectants (from 43.3% to 40% by DMSO/PROH, respectively), even DMSO group showed a slight increase in viability rate.

As you can see in figure 1, there is a downward trend from control group to 6-month group.

### Chromosome abnormality

**Fig 2:**
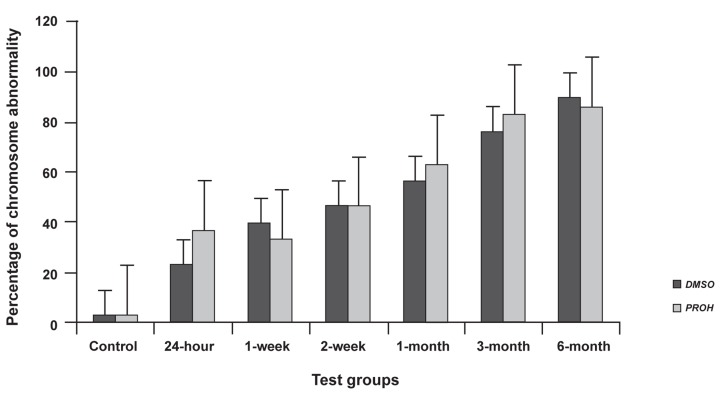
Percentage of chromosome abnormality at the presence of DMSO and PROH as cryoprotectant for various storage durations. Error bars show SE of mean values calculated for data obtained from different samples.

As a whole, about 360 embryos were subjected to cytological analysis. The results of cytological analysis are presented in figure 2 and table 2. Cryopreservation procedure resulted in greater than three-fold increase in the total level of mitotic abnormalities in both DMSO/PROH vitrified embryos from 24-hour group to 6-month group (24- hour group for DMSO and PROH showed 23.3% and 36.6%, respectively, but 6-month group for DMSO and PROH showed 90% and 86.6%, respectively).

In this study the total amount of chromosome abnormality, including aneuploidy and polyploidy are shown in figure 2. In this figure, there is an upward trend from control group to 6-month group, for both DMSO and PROH vitrified embryos (p values for all treatment groups were <0.05).

As indicated, increasing storage duration increased the incidence of chromosome abnormalities in all test groups compared to control group. It should be mentioned that it could be deduced from figure 2 and table 2 that DMSO was apparently a better cryoprotectant than PROH. Besides, it should be mentioned that 2-week groups of both cryoprotectants showed similar chromosome abnormalities.

**Table 2:** The results of total abnormalities of eight-cell mouse embryos vitrified in DMSO/PROH solution after various storage durations


Test groups	No. of Embryos					P value
	Vitrified	Intact	Chromosome abnormal embryos			
			Aneuploid *	Polyploid**	Percentage(%)	

Control	100	95	4	1	5(3)	<0.05
24-hour	30	23	3	4	7 (23.3)	<0.05
	30	19	6	5	11 (36.6)	
1-week	30	18	3	9	12(40)	<0.05
	30	20	7	3	10(33.3)	
2-week	30	16	10	4	14 (46.6)	<0.05
	30	16	9	5	14 (46.6)	
1-month	30	13	7	10	17 (56.6)	<0.05
	30	11	4	15	19 (63.3)	
3-month	30	7	6	17	23(76.6)	<0.05
	30	5	9	16	25(83.3)	
6-month	30	3	6	21	27(90)	<0.05
	30	4	3	23	26(86.6)	

* Aneuploid embryos; The embryos whose chromosome count was 37-43. ** Polyploid embryos; The embryos whose chromosome count was 70-84.

## Discussion

### Post – thaw viability

 order to evaluate post-thaw viability of vitrified eight-cell mouse embryos, we examined the effect of two different cryoprotectants (DMSO, PROH) in 6 different groups based on storage durations, including: 24-hour, 1-week, 2-week, 1-month, 3-month and 6-month groups. Survival rates, assessed by the developmental potential *in vitro*, showed variation in rang of 6.6% to 66.7%, depending on the cryoprotectant used and storage duration. The survival rates of vitrified embryos depend on several mechanisms of cell injury, such as the chemical toxicity of the cryoprotectant, intracellular ice formation, fracture damage, and osmotic swelling during the removal of the cryoprotectant.

In this study, we considered all the embryos with any kinds of injuries as degenerated embryos to evaluate the cryoprotectants as a whole. The same exposure time, cryoprotectant percentage and temperature were implemented, but the cryoprotectants and storage durations were different.

The freezing and warming of cells protrudes a series of pressures, such as equilibration with a cryoprotectant, cooling- warming, dilution and rehydration (31). As a cryoprotectant for conventional freezing of human embryos, PROH has been widely used; although, DMSO (32) has also proven effective.

According to Figure 1, we had poor post-thaw viability in those groups with longer storage durations. So far, there is many studies reporting good results with vitrification, but they had very short storage durations (33-35). In our study, the possible of poor rates of eight-cell embryos viability may be the result of storage duration which may be harmful for zona pellucida integrity. Vitale et al. (36) has suggested that excellent quality of frozen-thawed embryos at the eight- to 16-cell stage often do not develop *in vitro* without full protection of an intact zona pellucida. For early stage embryos, it is thought that the zona pellucida helps to maintain cellular integrity of the blastomeres. Our results may demonstrate the possibility of zona injury besides cryoprotectant toxicity that reduces postthaw viability. On the other hand, this viability reduction can be in charge of chemical toxicity of cryoprotectants due to increase of storage duration leading to damage of intra cellular components.

However, "freezing and thawing significantly reduces embryo viability" (12). The detrimental effects of cryopreservation may also result in damages to the cell membranes and intracellular components (37 , 38 ). Ideally, the freeze-thaw procedure should not cause any loss of viability, or lead to an increased incidence of genetic aberrations, fetal malformation or losses. An almost recent study from Belgium (7) including 547 cryo- ICSI and 390 cryo-IVF children has showed that cryo-ICSI twins has significantly higher preterm birth and very low birth weight rates than twins from fresh ICSI. Furthermore, a higher rate of malformations is noticed for cryo-ICSI as compared with fresh ICSI. Besides, in a recent study that compared the viability after cryopreservation, a lower viability of the embryos after vitrification was reported (39). In addition, in another meta-analysis of cryopreservation study, vitrifying mouse embryos was undertaken to determine the treatment effect of vitrification, and they also showed that treatment by vitrification decreased embryo viability compared with controls (40)

On the contrary, two large registry studies, one from Denmark (41) and the other one from France (42) showed no difference in malformation rates between cryo-children and children born after transfer.

For the almost newly introduced technique of vitrification, very limited data have been reported on post-thaw viability outcomes. "This emphasize the urgent need for properly controlled postthaw studies, follow-up studies of these embryos, and careful assessment of evidence currently available before this technique is added to daily routines" (43).

### Chromosome analysis

To the best of our knowledge, this is the first report describing the effect of storage duration on chromosomal situation of vitrified eight-cell mouse embryos at the presence of DMSO and PROH as cryoprotectant.

This study clearly demonstrated that increasing storage duration increases chromosome abnormalities. In addition, as shown in table 2, DMSO is almost a better cryoprotectant than PROH because of causing less chromosome aberrations.

In this study only the abnormalities, due to chromosome and mitotic apparatus damage have been investigated; in other word, we traced aneuploidy and polyploidy which the latter one occurred due to blastomere fusion. The lagging of whole chromosomes or their fragments are the most frequently detected mitotic abnormality due to chromosome damage. This phenomenon is due to damage of a kinetochore or loss of its function (44). Cryopreservation leads to fragmentation of chromosomes, thus increases the frequency of chromatid bridges arising in early and compacted embryos (45).

In contrast to our results, Bongso et al. (46) have demonstrated that cryopreservation of two-cell mouse embryos using DMSO or propanediol does not increase the incidence of aneuploidy or polyploidy.

It is known that the cytoskeleton of mammalian oocytes and embryos is sensitive to thermo-and chemo- stresses resulting from cryopreservation (47-49). Disorganization of the spindle after cryopreservation was observed in metaphases II oocytes and late two-cell embryos in mitosis (48). The damage of the mitotic apparatus after cryopreservation is confirmed initially by such gross disturbances as multipolar and unipolar mitosis; consequently, the unequal distribution of chromosomes between daughter cells results in aneuploidy (45). Moreover, Salumets et al. (50) has found a higher proportion of chaotic embryos after resumption of mitosis, followed by freezing and thawing of two-cell embryos. They; therefore, proposed that the freezing procedure can cause dysfunctional spindles.

In our study, polyploid embryos may be the result of blastomere fusion occurring due to possible zona injuries. Balakier et al. (51) has clearly showed that cryopreservation of early human embryos with standard propanediol technique may cause blastomere fusion in correlated with some existing membrane abnormalities that can result in fusion after freezing and thawing, leading to chromosomal aberrations.

Furthermore, in another study, Agerholm et al. (52) showed that at the time of freezing, none of the embryos had visible multinucleated blastomeres. After thawing, they found that 33% of the embryos were multinucleated. This suggests that the majority of the multinuclearity was introduced after thawing. Uncoupling of the processes that control karyokinesis and cytokinesis may result in binucleated blastomeres (53-55). Apparently, the data suggest that the freezing procedure could affect coupling of karyokinesis and cytokinesis which could; therefore, be result of the suboptimal conditions during the freezing procedure, resulting in a dysfunctional spindle (50).

From our present study, it is likely that freezing and thawing may be responsible for blastomere fusion. This observation is concordance with the obtained result of Balakier et al. (51). It may also indicate that blastomere fusion is not only because of fair and poor quality embryos, as was previously thought (1), but it can also alter embryos that are graded as "good morphology" group, as was shown by our observation (100% of affected embryos were of good quality).

Now, the question is how these vitrified embryos using in clinic can result in live and intact birth. It can also be analyzed from a different perspective: when an embryo is ready to be transferred, it must pass through certain barriers, such as cryopreservation, thawing, mitosis resumption, finally developmental obstacles; at the same time, it must be able to maintain its chromosomal status. For transferring a vitrified embryo, several other embryos may be lost due to lack of viability or chromosome abnormalities, and finally one lucky embryo passing through all of these risks is going to be transferred. More over, even after implantation, many of these embryos could be aborted because of chromosomal abnormality at the first trimester.

## Conclusion

It is probably premature to draw definite conclusions concerning the reasons of chromosomally abnormal embryos among frozen-thawed embryos, and our data strongly suggest that the majority of mitotic abnormalities in eight-cell mouse embryos may be consequences of damage to the mitotic apparatus and/or zona injury that could be due to the storage duration of cryopreserved embryos.

Our results may show that long-term cryopreservation that requires a long-term exposure of embryos to cryoprotectants, can cause low viability and/or chromosomal abnormalities.

While vitrification has a clear role in ART, the researches should continue to establish optimal vitrification method which may assist in alleviating concerns over safety issues, such as storage, transport and the use of very high cryoprotectant concentrations. In addition, analysis of global gene expression following cryopreservation and even DNA apoptosis may be successfully applied on excess human embryos.

## References

[1] Trounson A (1986). Preservation of human eggs and embryos. Fertil Steril.

[2] Bergh C, Werner C, Nilsson L, Hamberger L (1995). Cumulative
birth rates following cryopreservation of all
embryos in stimulated *in vitro* fertilization (IVF) cycles. J Assist Reprod Genet.

[3] Ombelet W, De Sutter P, Van der Elst J, Martens G (2005). Multiple gestation and infertility treatment: registration,
reflection and reaction-the Belgian project. Hum Reprod Update.

[4] Van Landuyt L, Verheyen G, Tournaye H, Camus M, Devroey P, Van Steirteghem A (2006). New Belgian embryo
transfer policy leads to sharp decrease in multiple
pregnancy rate. Reprod Biomed Online.

[5] Neubourg DD, Mangelschots K, Van Royen E, Vercruyssen M, Ryckaert G, Valkenburg M (2002). Impact
of patients' choice for single embryo transfer of
a top quality embryo versus double embryo transfer
in the first IVF/ICSI cycle. Hum Reprod.

[6] Gerris J, De Neubourg D, De Sutter P, Van Royen E, Mangelschots K, Vercruyssen M (2003). Cryopreservation
as a tool to reduce multiple birth. Reprod Biomed Online.

[7] Belva F, Henriet S, Van den Abbeel E, Camus M, Devroey P, Van der Elst J (2008). Neonatal outcome
of 937 children born after transfer of cryopreserved
embryos obtained by ICSI and IVF and comparison
with outcome data of fresh ICSI and IVF cycles. Hum Reprod.

[8] Li L, Zhang X, Zhao L, Xia X, Wang W (2012). Comparison
of DNA apoptosis in mouse and human blastocysts
after vitrification and slow freezing. Mol Reprod Dev.

[9] Fathi R, Valojerdi MR, Yazdi PE, Ebrahimi B, Alipour H, Hassani F (2012). Development of 4-cell mouse
embryos after re-vitrification. Cryobiology.

[10] Mochida K, Hasegawa A, Taguma K, Yoshiki A, Ogura A (2011). Cryopreservation of mouse embryos by
ethylene glycol-based vitrification. J Vis Exp.

[11] Liu WX, Lu H, Luo MJ, Xu LZ (2011). Effects of different
cryoprotectants and cryopreservation protocols on
the development of 2-4 cell mouse embryos. Cryo
Letters.

[12] Selick CE, Hofmann GE, Albano C, Horowitz GM, Copperman AB, Garrisi GJ (1995). Embryo quality
and pregnancy potential of fresh compared with frozen
embryos--is freezing detrimental to high quality
embryos?. Hum Reprod.

[13] Kito S, Noguchi Y, Ohta Y, Ohhata T, Abe M, Shiomi N (2003). Evaluation of developmental competence
of vitrified-warmed early cleavage stage embryos
and their application for chimeric mouse production. Exp Anim.

[14] Ezzatabadypour M, Hosseini A, Baharvand H, Nematolahi SN, Heydari MH (2002). Developmental potetial
of mouse morula early and late blastocyst after
vitrification. Cell J.

[15] Ramezani M, Rezazadeh Valojerdi M, Parivar K (2004). Comparison of the effects of different vitrification
methods on development of two-cell mouse embryos. Cell J.

[16] 1Wada I, Macnamee MC, Wick K, Bradfield JM, Brinsden PR (1994). Birth characteristics and perinatal
outcome of babies conceived from cryopreserved
embryos. Hum Reprod.

[17] Sutcliffe AG, D’souza SW, Cadman J, Richards B, McKinlay IA, Lieberman B (1995). Minor congenital anomalies,
major congenital malformations and development
in children conceived from cryopreserved
embryos. Hum Reprod.

[18] Sutcliffe AG, D’souza SW, Cadman J, Richards B, McKinlay IA, Lieberman B (1995). Outcome in children
from cryopreserved embryos. Arch Dis Child.

[19] Wennerholm UB, Hamberger L, Nilsson L, Wennergren M, Wikland M, Bergh C (1997). Obstetric and perinatal
outcome of children conceived from cryopreserved
embryos. Hum Reprod.

[20] Wennerholm UB, Albertsson-Wikland K, Bergh C, Hamberger L, Niklasson A, Nilsson L (1998). Postnatal
growth and health in children born after cryopreservation
as embryos. Lancet.

[21] Wennerholm UB, Bergh C, Hamberger L, Lundin K, Nilsson L, Wikland M (2000). Incidence of congenital
malformations in children born after ICSI. Hum Reprod.

[22] Stanger J, Wong J, Conceicao J, Yovich J (2012). Vitrification
of human embryos previously cryostored by
either slow freezing or vitrification results in high
pregnancy rates. Reprod Biomed Online.

[23] Edgar DH, Gook DA (2012). A critical appraisal of cryopreservation-
slow cooling versus vitrification-of human
oocytes and embryos. Hum Reprod Update.

[24] Quiroga R, Roselló M, Martinez F, Ferrer-Bolufer I, Monfort S, Oltra S (2009). Rare chromosomal complement
of trisomy 21 in a boy conceived by IVF and
cryopreservation. Reprod Biomed Online.

[25] Wood MJ (1997). Embryo freezing: is it safe?. Hum Reprod.

[26] Mozdarani H, Moradi SZ (2007). Effect of vitrification on
viability and chromosome abnormalities in 8-cell
mouse embryos at various storage durations. Bio Res.

[27] Ishida GM, Saito H, Ohta N, Takahashi T, Ito MM, Saito T (1997). The optimal equilibration time for
mouse embryos frozen by vitrification with trehalose. Hum Reprod.

[28] Mukaida T, Wada S, Takahashi K, Pedro PB, An TZ, Kasai M (1998). Vitrification of human embryos based
on the assessment of suitable conditions for 8-cell
mouse embryos. Hum Reprod.

[29] Kasai M (1997). Cryopreservation of mammalian embryos. Mol Biotechnol.

[30] Tarkovski AK (1996). An air drying method for chromosome
preparation from mouse eggs. Cytogenetics.

[31] Leibo SP (1992). Techniques for preservation of mammalian
germplasm. Anim Biotechnol.

[32] Van der Elst J, Camus M, Van den Abbeel E, Maes R, Devroey P, Van Steirteghem AC (1995). Prospective
randomized study on the cryopreservation of human
embryos with dimethylsulfoxide or 1, 2-propanediol
protocols. Fert Steril.

[33] Otsuka J, Takahashi A, Nagaoka M, Funabashi H (2002). Optimal equilibration conditions for practical vitrification
of two-cell mouse embryos. Comp Med.

[34] Bautista JA, Takahashi Y, Kanagawa H (1997). *in vitro* viability
of mouse 8-cell embryos vitrified in a simple
solution of ethylene glycol. Jpn J Vet Res.

[35] Bautista JA, Takahashi Y, Kanagawa H (1998). *in vitro* viability
of mouse zygote vitrified in ethylene glycol. Japanese Journal of Veterinary Research.

[36] Vitale NJ, Myers MW, Denniston RS, Leibo SP, Godke RA (1997). In-vitro development of refrozen mouse
embryos. Hum Reprod.

[37] Dumoulin JC, Bergers-Janssen JM, Pieters MH, Enginsu ME, Geraedts JP, Evers JL (1994). The protective
effects of polymers in the cryopreservation of
human and mouse zonae pellucidae and embryos. Fertil Steril.

[38] Ng SC, Sathananthan AH, Wong PC, Ratnam SS, Ho J, Mok H (1988). Fine structure of early human
embryos frozen with 1,2 propanediol. Gamete Res.

[39] AbdelHafez F, Xu J, Goldberg J, Desai N (2011). Vitrification
in open and closed carriers at different cell
stages: assessment of embryo survival, development,
DNA integrity and stability during vapor phase
storage for transport. BMC Biotechnol.

[40] Manno FA 3rd (2010). Cryopreservation of mouse embryos
by vitrification: a meta-analysis. Theriogenology.

[41] Pinborg A, Loft A, Rasmussen S (2008). Danish national
controlled cohort study on neonatal outcome of
1267 children born after transfer of cryopreserved
IVF and ICSI embryos in 1995 to 2006. 24^th^ Annual
Meeting of the ESHRE.

[42] Royere D, Levy R, Mouchel T (2006). Pregnancy issues after
frozen embryo transfer analysis based on 3632
pregnancies follow-up. 22^nd^ Annual Meeting of the
ESHRE.

[43] Van Steirteghem A (2008). What next for assisted reproductive
technology? A plea for an evidencebased
approach. Hum Reprod.

[44] Fiskejö G, O’Hare S, Atterwill AC (1995). Methods in molecular biology. Allium test.

[45] Khromenkova OB, Zhernoklev GV, Zhegunov GV, Grischenko VI (2003). The incidence of mitotic abnormalities
in cryopreserved eight-cell early and compacted
mouse embryos. Cryo Letters.

[46] Bongso A, Chye NS, Sathananthan H, Mui-Nee L, Mok H, Wong PC (1988). Chromosome analysis of
two-cell mouse embryos frozen by slow and ultrarapid
methods using two different cryoprotectants. Fertil Steril.

[47] Pickering SJ, Johnson MH (1987). The influence of cooling
on the organization of the meiotic spindle of the
mouse oocyte. Hum Reprod.

[48] Sathananthan AH, Ng SC, Trounson AO, Bongso A, Ratnam SS, Ho J (1988). The effects of ultrarapid
freezing on meiotic and mitotic spindles of mouse
oocytes and embryos. Gamete Res.

[49] Van der Elst J, Van den Abbeel E, Jacobs R, Wisse E, Van Steirteghem A (1988). Effect of 1, 2-propanediol and dimethylsulphoxide
on the meiotic spindle of the mouse
oocyte. Hum Reprod.

[50] Salumets A, Horelli-Kuitunen N, Suikkari AM, Metspalu A, Tuuri T (2004). Elevated incidence of chromosomally
chaotic embryos among frozen-thawed preimplantation
embryos. Eur J Obstet Gynecol Reprod Biol.

[51] Balakier H, Cabaca O, Bouman D, Shewchuk AB, Laskin C, Squire JA (2000). Spontaneous blastomere fusion
after freezing and thawing of early human embryos
leads to polyploidy and chromosomal mosaicism. Hum Reprod.

[52] Agerholm IE, Kølvraa S, Crüger DG, Berg C, Bruun-Petersen G, Ziebe S (2008). Resumption of mitosis in
frozen-thawed embryos is not related to the chromosomal
constitution. Fertil Steril.

[53] Hardy K, Winston RM, Handyside AH (1993). Binucleate
blastomeres in preimplantation human embryos *in vitro*: failure of cytokinesis during early cleavage. J Reprod Fertil.

[54] Pickering SJ, Taylor A, Johnson MH, Braude PR (1995). An
analysis of multinucleated blastomere formation in
human embryos. Hum Reprod.

[55] Hnida C, Engenheiro E, Ziebe S (2004). Computer-controlled,
multilevel, morphometric analysis of blastomere
size as biomarker of fragmentation and multinuclearity
in human embryos. Hum Reprod.

